# The Behavioural Response of Australian Fur Seals to Motor Boat Noise

**DOI:** 10.1371/journal.pone.0037228

**Published:** 2012-05-18

**Authors:** Joy S. Tripovich, Sophie Hall-Aspland, Isabelle Charrier, John P. Y. Arnould

**Affiliations:** 1 Faculty of Veterinary Science, University of Sydney, Sydney, New South Wales, Australia; 2 Institute of Marine and Antarctic Studies, University of Tasmania, Hobart, Tasmania, Australia; 3 Centre de Neurosciences, Université Paris Sud, Paris-Sud, Orsay, France; 4 School of Life and Environmental Sciences, Deakin University, Burwood, Victoria, Australia; University of Sussex, United Kingdom

## Abstract

Australian fur seals breed on thirteen islands located in the Bass Strait, Australia. Land access to these islands is restricted, minimising human presence but boat access is still permissible with limitations on approach distances. Thirty-two controlled noise exposure experiments were conducted on breeding Australian fur seals to determine their behavioural response to controlled in-air motor boat noise on Kanowna Island (39°10′S, 146°18′E). Our results show there were significant differences in the seals' behaviour at low (64–70 dB) versus high (75–85 dB) sound levels, with seals orientating themselves towards or physically moving away from the louder boat noise at three different sound levels. Furthermore, seals responded more aggressively with one another and were more alert when they heard louder boat noise. Australian fur seals demonstrated plasticity in their vocal responses to boat noise with calls being significantly different between the various sound intensities and barks tending to get faster as the boat noise got louder. These results suggest that Australian fur seals on Kanowna Island show behavioural disturbance to high level boat noise. Consequently, it is recommended that an appropriate level of received boat sound emissions at breeding fur seal colonies be below 74 dB and that these findings be taken into account when evaluating appropriate approach distances and speed limits for boats.

## Introduction

Anthropogenic disturbance is a significant catalyst of environmental change, with potentially important implications for individuals and populations [1]. There is compelling evidence supporting an association between stress in mammals, in terms of their physiology and behaviour, with human disturbance [1], [2], [3]. Human disturbance was reported to negatively influence the breeding success in penguins [2], while in brown bears (*Ursus arctos*) human disturbance was found to increase energetic expenditure as a result of behavioural modifications in the species [3]. Anthropogenic disturbance has also been associated with changes in the spatial distribution of hyena clans [4]. Fundamentally, any modifications to biologically significant activities such as an animal's hormonal state, behaviour and energy reserves may ultimately affect an animal's fitness and have detrimental effects on the population [1].

Australian fur seals, (*Arctocephalus pusillus doriferus*), are endemic to Australia and breed on thirteen islands located in south-eastern Bass Strait [5]. These breeding islands provide areas where seals give birth, mate and raise their young away from potential threats of terrestrial predators, including humans. During the breeding season, male Australian fur seals compete aggressively to establish and maintain territories. At this time, females give birth, mate shortly thereafter and then alternate between feeding out at sea and suckling their young on land [6]. Vocal communication, olfaction and postural displays are intrinsic to the breeding behaviour of Australian fur seals [6]. Therefore, the use of sight, smell and hearing are critically important to the species' ecological and breeding strategy.

The breeding season is an energetically costly period for Australian fur seals, where males generally fast during territorial tenure and females require energy to give birth, forage and suckle their young. Consequently, any disturbance such as anthropogenic noise from boats that induces energetically costly behaviours such fleeing or activities that interfere with communication during this critical time may detract from energy reserves that are required for breeding.

Human disturbance is a key threat to seals in Australia (EPBC Act 1999) [7] with little or no quantitative data available on the impact of anthropogenic noise on seals in the Southern Hemisphere. In Australian fur seals, there have been three studies evaluating the response of seals to boat disturbance. Two studies conducted at the haul-out sites of Steamers Head (35°10′S, 150°40′E) [8] and Montague Island (36°20′S, 150°10′E) [9] reported that the responses of Australian fur seals to boat-based approaches included increased vigilance and fleeing behaviours. Back [10] conducted controlled boat approaches at two breeding sites (Kanowna Island and Seal Rocks, 39°10′S, 146°18′E) and found similar fleeing behaviours, but also observed that seals at these two breeding colonies had markedly different responses, presumably due to habituation. Shaughnessy et al. [9] further commented that it is unclear as to whether the seals are reacting to boats due to changes in sight, sound or odour. As these boat approach studies did not control for the impact of noise alone, this instigated the current project, whereby we conduct quantitative studies examining the effect of noise generated from human disturbance on seals' behaviour.

Anthropogenic noise exposure has been associated with stampedes and the crushing or abandonment of pups [11]. Many studies have reported pup mortality associated with abandonment by female pinnipeds during disturbance events, such as aircraft overflights (e.g., [12], [13]). Burleigh et al. [8] reported that stampedes at fur seals haul-out sites occurred in response to boat approaches at 20 m, and stampedes have also been reported at Kanowna Island due to boat approaches [14]. The challenge posed by disturbance studies is determining the appropriate or acceptable levels of disturbance. Therefore, the present study aims to investigate the behavioural and vocal response of breeding Australian fur seals at Kanowna Island to anthropogenic disturbance, through controlled noise exposure experiments.

## Materials and Methods

### Ethics Statement

Research for this project was conducted under Ethics Number A10/2008, Animal Welfare Committee, Deakin University.

### Study site

The study examined the behavioural response of Australian fur seals on Kanowna Island (39°10′S, 146°18′E), Bass Strait, Australia, during the November–December 2008 breeding season over a two week period. The island has two main colonies (East Valley and Main Colony) and these comprised males, females, yearlings and pups at the time of sampling.

Kanowna Island is situated within a Marine National Park and access to the island is restricted, with boat-based approaches being permissible under seasonal contingent minimum distances of 200 m during the breeding season (November to January) and 50 m during the non-breeding season (February to October) [15].

### Noise Playback Experiment

A total of 112 animals were exposed to a range of randomly selected in-air motor boat noise, played at three different sound intensities: 1) Low 64–70 dB (n = 27 animals); 2) Mid 71–74 dB (n = 49 animals); and 3) High 75–85 dB (n = 36 animals). These are perceived levels, so this is the intensity received by seals at the centre of each group under study. The range of intensity for each level is explained because of the spatial spread of animals within each group. The signals were broadcast at 5–15 m from the subjects but the sound emission was calibrated at each playback to ensure the received levels were known, see below. Thirty-two experiments were conducted on groups of seals ranging from 1 to 11 animals (average = 4 animals) and consisted of three phases: pre-stimulus (10 min), stimulus (2 min) and post-stimulus (10 min). Each group of seals, i.e. 32 groups, were used once in each experiment, so were either played the low, mid or high sound levels, and the choice of each treatment used was randomised.

**Figure 1:**
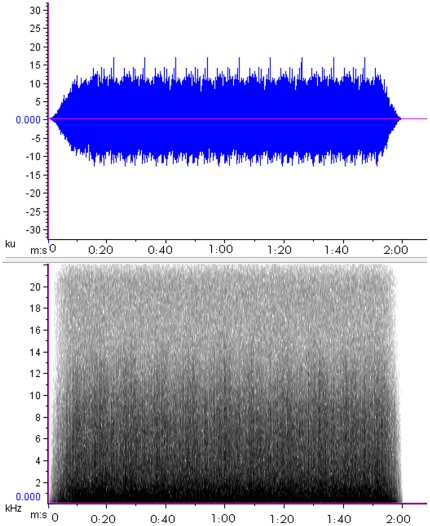
An example of acoustic features of boat noise used in controlled noise experiments on Kanowna Island. Top Panel is motor boat noise showing the amplitude of the boat noise and Lower Panel is the spectrogram of motor boat noise, spectrogram parameters: 256-point FFT, 256-point Hanning window with 50% overlap.

The motor boat noise used in the playbacks (Fig. 1) was pre-recorded from a range of boats (n = 7). For the purpose of this study, the sound intensity emitted by each vessel was standardised to focus on examining the effects of varying sound intensities only.

Playbacks were broadcast using a MIPRO707 speaker (frequency response 60 Hz–20,000 kHz±3 dB). The speaker was connected to a Sony digital tape recorder (TCD-D8; Sony Corp., Japan) and placed 5–15 m away from subjects. Several propagation tests were performed on the island to ensure that the received sound pressure was known. The sound pressure was measured with a Radio Shack Model 33–2050 sound level meter set at “C” weighting and fast response. Duration between two trials was at least 5 h to reduce any habituation effect by the animals in the colony.

### Evaluation of responses

Four different methods were used to evaluate the responses of seals (Table 1).

1. Behavioural scan sampling to determine behaviours pre-stimulus, stimulus and post-stimulus – Video recordings were used to analyse the behaviours of males, females, yearlings and pups (Table 2). A scan sampling regime was used [16] where the behaviours of individual seals were recorded every 30 s for the duration of the experiment. Behaviours were grouped according to one of three phases: pre-stimulus, stimulus and post-stimulus and each category of behaviour was analysed separately over the three phases to determine significant changes in behaviour between the phases.
2. Behavioural response during stimulus phase – The behavioural response of seals was examined to determine whether seals responded differently to the three received levels of motor boat noise. This experiment contained three treatments: low boat noise (64–70 dB); mid boat noise level (71–74 dB); and high level motor boat noise (75–85 dB). Individual seals behavioural responses observed during the noise playback (i.e. stimulus phase) was analysed. Each cohort's (i.e. males, females, yearlings and pups) response to the playbacks was graded on an ordinal scale from 0 to 3. The scale was as follows: 0 was no response; 1 was eye movements towards the noise source; 2 head and/or body movements towards the noise source; and 3 movements away from the noise source.
3. The length of time seals looked at the noise source – The time a seal spent looking in the direction of the noise during the stimulus phase (2 min in duration) was recorded, and compared between individual seals over the three sound intensities.
4. Acoustic behaviour of seals during the pre and post-stimulus phase – As males are the most vocal age group they were chosen for the sound analysis (See [6], [17]) for details on analysis procedure). Vocalisations were analysed using RAVEN Pro 1.4 (Ithaca, NY) for the pre-stimulus and post-stimulus phase, but were not analysed for the stimulus phase as the recordings were of poor quality due to the boat vessel noise emitted during the stimulus phase. Twenty randomly selected bark calls from eleven males were analysed from the pre and post-stimulus phases. Only temporal features such as the repetition rate, unit and inter-unit duration were examined for changes in call structure with changes in noise levels.

**Table 1:** Description of evaluation methods to analyse the response of Australian fur seals during the noise playback experiments.

Method	Description
*1. Behavioural responses during pre-stimulus, stimulus and post stimulus phases*	Behaviours were grouped according to one of three phases: pre-stimulus, stimulus and post-stimulus and each category of behaviour was analysed separately over the three phases to determine significant changes in behaviour between the phases.
*2. Behavioural response during stimulus phase*	Individual seals behavioural responses observed during the noise playback (i.e. stimulus phase) was analysed.
*3. The length of time seals looked at the noise source during the stimulus phase*	The time a seal spent looking in the direction of the noise during the stimulus phase (2 min in duration) was recorded, and compared between individual seals over the three sound intensities.
*4. Acoustic response during pre and post stimulus*	Vocalisations were analysed for the pre-stimulus and post-stimulus phase

**Table 2:** Ethogram of Australian fur seal behaviours on Kanowna Island during playback study.

Behaviour	Definition
Rest	The seal positions itself with either the ventral or lateral surface of its torso against the substrate. The head is raised slightly when vocalising
Alert	The seal is sitting in an upright posture, where the subject looks towards the noise source
Fighting	This includes, open mouth threat, chasing, lunging and biting
Locomotion	The male usually moves forward and he waves his neck from side to side, this behaviour is often accompanied with vocalisation as it moves forward, but it remains within its territory
Obstructing	The seal impedes the movement of another by using its body as an obstruction
Nursing	Pup is suckling its mother's teat
Nuzzling	Where the vocalising animal touches the muzzle, nape or any other part of another seals body, using its muzzle. This behaviour may occur in water or on land, and generally occurs between a male and female seal or between mother and pup

### Statistical Analysis

General linear mixed model analysis was performed in GENSTAT (Release 13.1, U.K.) to determine significant changes in behaviour between the three phases (pre-, stimulus and post-stimulus). The fixed terms in the model were the phases (pre-, stimulus, and post-stimulus), the cohort (which was later removed) and the sound level (low, mid and high) with the random term as the animal ID. Each behaviour was examined separately as the dependent variable with the three phases as the independent variable. As there were no overall differences in the responses of the cohort (χ^2^ = 5.70, DF = 3, P = 0.127) to the three treatments (low, mid, high sound levels) it was taken out of the model.

The behavioural response during the stimulus were analysed using the ordinal logistic regression analysis using MINITAB Version 15 (MINITAB INC, USA). The behavioural response (coded as 0 was no response; 1 was eye movements towards the noise source; 2 head and/or body movements towards the noise source; and 3 movements away from the noise source) was used as the dependent variables and was compared to the sound levels (low, mid and high) which were the independent variables included in the model. This analysis established the significance of any differences between the variables (i.e., low, mid, high) and determined a probability of each behaviour response for each treatment [see 18]. Results were considered significant at *P*<0.05.

A uni-variate generalised linear model (GLM) was used to determine if the time a seal spent looking in the direction of the sound source (i.e. dependent variable) differed significantly between sound levels (i.e., low, mid, high) using SPSS (PASW Statistics 18, USA).

A multivariate GLM was performed to determine the difference in the dependent variable, the call characteristics (i.e., call duration, inter-unit duration and repetition rate-dependent variable) and the independent variables: the pre and post-stimulus phases, at the three different sound levels (low, mid and high). A Bonferroni correction was applied to the results of the tests to reduce the effect of increasing the probability of a Type I error.

## Results

### Behavioural responses during pre-stimulus, stimulus and post stimulus phases

Individual behaviours (Table 2) were compared among three phases (i.e. pre-stimulus, stimulus, post-stimulus) using generalised mixed model (GLMM) analysis, with 112 individual seals used in this analysis. There were significant differences in only three behaviours: Rest (P<0.001), alert (P<0.001) and fight (P<0.001) over the three phases. The rest behaviour was lowest during the stimulus phase, whilst the alert and fighting behaviours were highest during this period. Seals were most alert during the stimulus but this decreased during the post-stimulus phase. The opposite was observed for the resting behaviours. Resting was reduced during the stimulus phase but increased during the post-stimulus phase. Fighting was highest during the stimulus phase and decreased slightly during the post-stimulus phase but remained higher than the pre-stimulus phase.

### Behavioural response during stimulus phase

**Figure 2:**
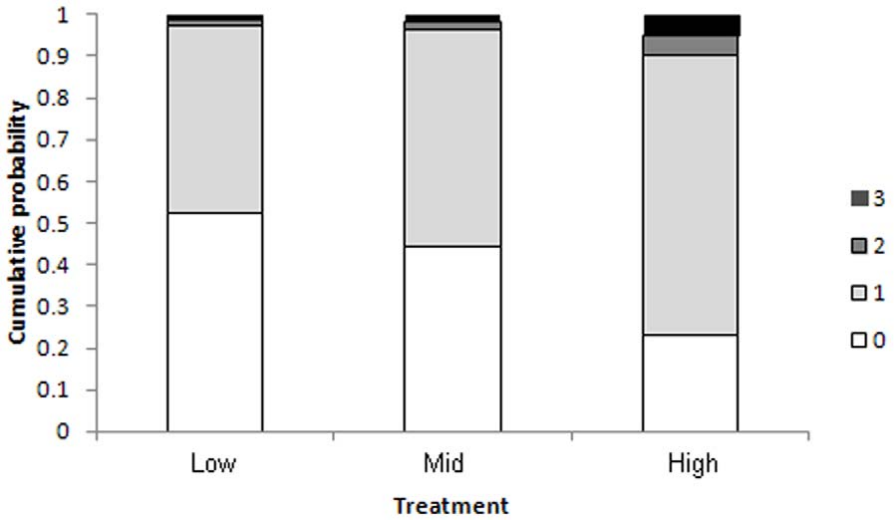
Fitted multinomial probabilities from the maximum likelihood of the proportional odds model from the ordinal logistic analysis. Where 0 = no response; 1 = eye movements towards the sound source; 2 = body movement towards the sound source; and 3 = move away from the sound source. The four shaded areas of the stacked bar chart represent the fitted probabilities of the four responses (0, 1, 2 & 3) for each treatment. 0 is represented in white, 1 in light grey and these represent minimal or no responses, 2 is represented by dark grey and 3 in Black and these represent the stronger responses of seals hearing the boat noise.

There were significant differences between the responses of individual seals (i.e. 112 individual seals from 32 playback experiments) to the different noise levels (χ^2^ = 8.14, DF = 2, P = 0.017). Seals did not respond significantly differently to the low and mid sound level treatment (Z = −0.67, P = 0.504) and mid *versus* high sound levels (Z = −1.87, P = 0.062) but there were significant differences in the response of seals hearing the low *versus* high sound levels (Z = −2.83, P = 0.005). Seals reacted more strongly (i.e. moved body in the direction of the noise source or physically moved away) in response to hearing the louder sounds (Fig. 2).

### The length of time seals looked at the sound source during the stimulus phase

The univariate GLM found that from 112 seals examined that the time they spent looking towards the noise source showed a strong tendency towards being significant (F = 2.99, P = 0.05). Overall seals spent more time looking at the noise source during high level disturbances.

### Acoustic response during pre and post stimulus

A multivariate GLM was performed on eleven male seals comparing each of the acoustic properties analysed (i.e. unit, inter-unit duration and repetition rate) for both pre and post-stimulus at the three sound intensities (i.e. low, mid and high). There were statistically significant differences in male call parameters between the pre- and post-stimulus phases (*F*_3, 398_ = 5.46, P = 0.01; Wilk's Lambda = 0.960). There were also significant differences in the calls at the different sound levels measured during the pre- and post-stimulus phases, (*F*_6, 796_ = 14.24, P = 0.001; Wilk's Lambda = 0.816). Post-hoc tests for each call feature measured demonstrates that the unit-duration of bark calls is significantly different between the low and mid-level treatment (P = 0.002), and there are significant differences for the inter-unit duration between low and mid (P<0.001) and low and high-level (P = 0.004). Overall, both the unit and inter-unit duration got longer as the sound level increased (Table 3). The repetition rate of barks varied significantly between treatment low and high level (P = 0.049), and mid and high levels (P = 0.011), getting faster as the sound got louder (Table 4).

**Table 3:** Mean and SE values for the acoustic parameters of male Australian fur seal bark calls during the playback experiments.

Call feature			Mean	SE			Mean	SE
Unit duration	Pre	Low	133.8	4.2	Post	Low	127.8	4.0
(msec)		Med	136.9	3.3		Med	138.1	4.5
		High	142.6	4.0		High	150.1	3.3
Inter-unit duration	Pre	Low	202.5	6.4	Post	Low	199.8	6.2
(msec)		Med	224.2	6.2		Med	218.5	6.8
		High	241.8	5.0		High	223.6	5.0
Repetition rate	Pre	Low	2.9	0.1	Post	Low	3.4	0.1
(barks units/sec)		Med	3.3	0.1		Med	3.4	0.1
		High	3.1	0.1		High	3.2	0.1

**Table 4:** Post-hoc comparisons for the acoustic parameters of male Australian fur seal bark calls during the playback experiments.

Call feature	Comparison	P
Unit duration	Low vs Med	0.002
	Low vs High	0.056
	Med vs High	1.000
Inter-unit duration	Low vs Med	<0.001
	Low vs High	0.004
	Med vs High	0.141
Repetition rate	Low vs Med	1.000
	Low vs High	0.049
	Med vs High	0.011

(* indicates significance at P<0.05).

## Discussion

The study reports the behavioural response of breeding Australian fur seals to motor boat noise. Using controlled noise exposure experiments we were able to quantify the response of seals to three sound intensities to determine a relatively safe received level of boat noise below 74 dB. Our findings further reveal that Australian fur seals utilize vocal plasticity to cope with changes in anthropogenic noise. The results suggest seals perceive boats as potential threats with louder motor boat noise having a greater impact on seals, where seals displayed more aggressive and alert behaviours.

In the present study, seals reacted more strongly to hearing the louder sounds by either orientating themselves towards the boat noise or physically moving away. Seals displayed significantly different responses between low (64–70 dB) and high levels (75–85 dB), indicating that in air, noise levels above 74 dB are predicted to cause behavioural disturbance in Australian fur seals. At high levels (75–85 dB), seals displayed energetically costly behaviours. During one of the playback experiments (high level), seals began to move rapidly away from the noise, displaying a cascading effect resembling those in the initial stages of a stampede. The playback was immediately stopped to reduce the potential for seals, particularly pups, to get crushed, injured or killed. Our findings from the behavioural response of seals support the notion that received boat noise levels at 75–85 dB elicit significant aversive behaviours by seals which may potentially cause injury.

Many species of animals are heavily reliant on acoustic signals for intra-specific communication (birds: [19], for review on marine mammals see [20]. Environmental selection pressures such as background noise may affect how well vocal features such as repertoire size, spectral and temporal characteristics are communicated [21]. Vocalising animals have access to a number of signalling strategies to avoid or reduce masking by ambient noise (birds: [22]; marine mammals: [23]. Animals follow the mathematical theory of communication [24] whereby under noisy conditions animals alter their frequencies, vocalise more often, with longerand louder calls in order to communicate the same meaning and volume of information [25], [26], [27], [28], [29], [30]; in marine mammals: [23], [31], [32], [33]. Changes in vocalizations produced by Australian fur seals were evident, where seals increased their repetition rate to adjust to changes in anthropogenic noise. Seals continued to show signs of this behavioural modification during the post-stimulus phase, where vocalisations had not returned to their pre-stimulus ‘baseline’ calling pattern.

There were also significant differences in the call structure with varying levels of noise exposure. Other studies analysing the behavioural context of the bark call in male Australian fur seals have indicated that the rate of delivery increases when seals display intra-species aggression or when trying to ‘herd’ females [6]. It has been suggested that varying the acoustic structure of the bark may update recipients on the emotive state of the caller [34], [35] and this may be important information for researchers to understand. Changes in the acoustic structure of calls, particularly increasing the rate of delivery of the bark, indicate potential changes in emotion resembling more aggressive or heightened behaviours [36], [37], [38]. Therefore, when higher intensities of noise were played to seals and they produced vocalisations with faster repetition rates than would ordinarily be produced to signify aggressive responses, that this may in fact communicate a heightened sense of emotion in seals. This change in behaviour seems likely to indicate that seals recognise motor boat noise as a potential threat.

The current study did not examine if the noise exposure modified the amplitude or frequency spectrum of the calls, as this could potentially be shifted. For example, North Atlantic right whales were found to adjust the volume (i.e. amplitude) of their song in increased background noise, singing louder during higher levels of background noise [26]. Another way to cope to changing noise conditions is by modifying the frequency characteristics of vocalisations. Indo-Pacific Bottlenose Dolphins produced whistles of lower frequencies with fewer frequency modulations whereas dolphins living in habitats with less ambient noise, they produced whistles at varying frequencies with greater modulations [33]. In humpback whales, males produced longer songs during LFA sonar transmissions to compensate for acoustic interference [32]. Further investigations should be done to examine the other potential changes in the vocalisations of Australian fur seals.

Access is not permitted within 50 metres of Kanowna Island between February to October (inclusive), and for the protection of breeding Australian fur seals, access is not permitted within 200 metres of Kanowna Island during the breeding season between November to January (inclusive) [15]. The results of this study suggest that a safe received level is less than 74 dB. It is important to understand that a range of factors affect the actual received levels of sound at a colony including the boat speed, engine size, wind speed and direction and other factors such as smell and sight of the boat may affect the behavioural responses. Therefore, further propagation tests are required to convert the acceptable level of 74 dB of motor boat noise into distance approaches. These propagation tests should be conducted with a range of boat sizes, engine, wind speed, wind direction and boat speeds to determine the range of variables suitable to ensure the 74 dB sound intensity is not exceeded at breeding colonies. Back [10] suggested that approach distances at naïve colonies, i.e. colonies that have not been exposed to a lot of disturbance, such as Kanowna Island should be extended to >75 m to reduce disturbance of seals by boats. Burleigh et al. [8] also recommends that an approach distance of at least 75 m is warranted from haul-outs when there are fewer than 50 seals and at least I00 metres when there are 50 or more seals. In addition to studies that measure source levels of various types of boats at different speeds, noise propagation tests should be conducted to determine the minimum approach distance whereby noise levels would not exceed 74 dB, which is known to cause more aggressive and alert behaviours in breeding fur seals.

## References

[1] French SS, Gonzalez-Suarez M, Young JK, Durham S, Geber LR (2011). Human Disturbance Influences Reproductive Success and Growth Rate in California Sea Lions (Zalophus californianus)..

[2] Giese M, Handsworth R, Stephenson R (1996). Measuring Resting Heart Rates in Penguins Using an Artificial Egg (Midiendo Ritmos Cardiacos de Pingüinos (Sphenisciformes) en Reposo Utilizando un Huevo Artificial)..

[3] Rode KD, Farley SD, Fortin J, Robbins CT (2007). Nutritional consequences of experimentally introduced tourism in brown bears. J. Wildl. Manag..

[4] Boydston EE, Kapheim KM, Watts HE, Szykman M, Holekamp KE (2003). Altered behaviour in spotted hyenas associated with increased human activity.. Animal Conservation.

[5] Kirkwood R, Pemberton D, Gales R, Hoskins AJ, Mitchell T (2010). Continued population recovery by Australian fur seals. Mar. Freshw. Res..

[6] Tripovich JS, Canfield R, Rogers TL, Arnould JPY (2008). Characterization of Australian fur seal vocalizations during the breeding season..

[7] Environment Protection and Biodiversity Conservation Act (1999). Office of Legislative Drafting and Publishing, Attorney-General's Department, Canberra, Australia.

[8] Burleigh A, Lynch T, Rogers T (2008). ‘Best practice techniques for monitoring the fur seal haul-out site at Steamers Head, NSW, Australia’. Too close for comfort: contentious issues in human-wildlife encounters, edited by Daniel Lunney, Adam Munn and Will Meikle, 2008.

[9] Shaughnessy PD, Nicholls AO, Briggs SV (2008). Do tour boats affect fur seals at Montague Island, New South Wales?.

[10] Back J (2010). Behavioural responses of Australian fur seals to boat-based ecotourism..

[11] Holst M, Greene CR, Richardson WJ, McDonald TL, Bay K (2011). Responses of Pinnipeds to Navy Missile Launches at San Nicolas Island, California 37.

[12] Johnson BW (1977). The effects of human disturbance on a population of harbor seals..

[13] Wickens PA, Shelton PA, David JHM, Field JG, Oosthuizen WH (1992). A fur seal simulation model to explore alternative management strategies..

[14] Patkin K (2005). The sustainability of interactions between pinniped and marine users: A case study of Kanowna Island, Wilson's Promontory Marine National Park..

[15] Wilsons Promontory Marine National Park (2006). Wilsons Promontory Marine National Park, Management Plan, Victoria Australia..

[16] Altmann J (1974). Observational study of behavior: sampling methods..

[17] Tripovich JS, Rogers TL, Arnould JPY (2005). Species-specific characteristics and individual variation of the Bark Call produced by male Australianfur seals (Arctocephalus pusillus doriferus). Bioacoust..

[18] Dobson AJ (2001). An Introduction to Generalized Linear Models, second edition..

[19] Marler P, Slabbekoorn H (2004). Nature's music..

[20] Tyack PL (1999). Communication and Cognition. In: Biology of Marine Mammals (Reynolds III J. E. & Rommel S. E., eds).. Smithsonian Institution Press, Washington DC, pp.

[21] Slabberkoorn H, Ripmeester EAP (2008). Birdsong and anthropogenic noise: implications and applications for conservation. Mol. Ecol..

[22] Brumm H, Slabbekoorn H (2005). Acoustic communication in noise. Adv. in the Study of Behav..

[23] Parks S, Johnson M, Nowacek D, Tyack PL (2011). Individual right whales call louder in increased environmental noise.. Biology Letters.

[24] Shannon CE, Weaver W (1949). The Mathematical Theory of Communication..

[25] Bermúdez-Cuamatzin E, Ríos-Chelén AA, Gil D, Macías Garcia C (2010). Experimental evidence for real-time song frequency shift in response to urban noise in a passerine bird. Biol.Lett.. July 7,.

[26] Brumm H (2004). The impact of environmental noise on song amplitude in a territorial bird. J. Anim. Ecol..

[27] Slabberkoorn H, Peet M (2003). Birds sing at a higher pitch in urban noise.. Nat.

[28] Pytte CL, Rusch KM, Ficken MS (2003). Regulation of vocal amplitude by the blue-throated hummingbird, Lampornis clemenciae. Anim. Behav..

[29] Penna M, Hamilton-West C (2007). Susceptibility of evoked vocal responses to noise exposure in a frog of the temperate austral forest. Anim. Behav..

[30] Nemeth E, Brumm H (2009). Blackbirds sing higher pitched songs in cities: adaptation to habitat acoustics or side effect of urbanization?. Animal Behaviour.

[31] Fristrup KM, Hatch LT, Clark CW (2003). Variation in humpback whale (Megaptera novaeangliae) song length in relation to low-frequency sound broadcasts. J. Acoust. Soc. Am..

[32] Miller PJO, Biassoni N, Samuels A, Tyack PL (2000). Whale songs lengthen in response to sonar.. Nature.

[33] Morisaka T, Shinohara M, Nakahara F, Akamatsu T (2009). Effects of ambient noise on the whistles of Indo-Pacific bottlenose dolphin populations. J. Mammal..

[34] Schusterman RJ (1977). Temporal patterning in sea lion barking (Zalophus californianus). Behav. Biol..

[35] Miller EH (1991). Communication in pinnipeds, with special reference to nonacoustic signalling.. Behaviour of Pinnipeds (D.

[36] Fernández-Juricic E, Campagna C, Enriquez V, Ortiz CL (2001). Vocal rates and social context in male South American sea lions. Mar. Mamm. Sci..

[37] Kunc HP, Wolf JBW (2008). Seasonal changes of vocal rates and their relation to territorial status in male Galápagos sea lions (Zalophus wollebaeki). Ethol..

[38] Charrier I, Ahonen H, Harcourt RG (2011). What Makes an Australian Sea Lion (Neophoca cinerea) Male's Bark Threatening? J. Comp. Psych..

